# Curiosity Search: Producing Generalists by Encouraging Individuals to Continually Explore and Acquire Skills throughout Their Lifetime

**DOI:** 10.1371/journal.pone.0162235

**Published:** 2016-09-02

**Authors:** Christopher Stanton, Jeff Clune

**Affiliations:** Computer Science Department, University of Wyoming, Laramie, Wyoming, United States of America; University of Vermont, UNITED STATES

## Abstract

Natural animals are renowned for their ability to acquire a diverse and general skill set over the course of their lifetime. However, research in artificial intelligence has yet to produce agents that acquire all or even most of the available skills in non-trivial environments. One candidate algorithm for encouraging the production of such individuals is Novelty Search, which pressures organisms to exhibit different behaviors from other individuals. However, we hypothesized that Novelty Search would produce sub-populations of specialists, in which each individual possesses a subset of skills, but no one organism acquires all or most of the skills. In this paper, we propose a new algorithm called Curiosity Search, which is designed to produce individuals that acquire as many skills as possible during their lifetime. We show that in a multiple-skill maze environment, Curiosity Search does produce individuals that explore their entire domain, while a traditional implementation of Novelty Search produces specialists. However, we reveal that when modified to encourage intra-life behavioral diversity, Novelty Search can produce organisms that explore almost as much of their environment as Curiosity Search, although Curiosity Search retains a significant performance edge. Finally, we show that Curiosity Search is a useful helper objective when combined with Novelty Search, producing individuals that acquire significantly more skills than either algorithm alone.

## Introduction

Evolutionary Algorithms (EAs) have proven useful in a variety of fields, including space exploration [1], automated damage recovery [2], and evolving gaits for robots [3, 4]. While EAs often outperform human engineers [4, 5], the individuals they produce are usually *specialists*; in other words, they rarely possess more than a small subset of skills and are typically suited to only one task. However, we do not want a fleet of thousands of different software or robot agents that each only perform one task; instead, we want to produce *generalists* that can learn to perform a wide variety of different, useful skills over time [6, 7]. Additionally, the more generally capable agents are, the more likely they will be able to adapt to novel situations or changes in their environment [8–10].

Natural animals are a prime example of generalists, acquiring a large and diverse skill set over the course of their lifetime [11–13]. These skills allow animals to adapt to a wide range of unforeseen circumstances for which they were not originally evolved. For example, urban coyotes can learn how to cool themselves by sneaking into refrigerators [13]; cheetahs are known to use cars as vantage points for hunting [11]; and chimpanzees will stack boxes to obtain a banana otherwise out of their reach [12]. Ultimately, we would like EAs to foster the same level of adaptability in the artificial agents they produce.

One theory for how natural animals become generalists is that they are intrinsically motivated to explore their environment, e.g. through curiosity [14–17], which leads to the acquisition of many diverse skills. For example, many juvenile animals spend much of their time playing, which is thought to facilitate their ability to learn skills for surviving in unforeseen, stressful, or chaotic situations [8]. Curiosity motivates play; animals often play with objects they do not understand, experiment with behaviors they have not yet mastered, and sometimes deliberately seek out unexpected events to increase their understanding and survivability [8, 14]. Although the intrinsic motivation to explore has been imitated in artificial agents via machine learning [7, 18], the production of generalists within an evolutionary context is a relatively unexplored (and unsolved) problem. The skills discovered by EAs may be diverse, but are frequently fragmented across different members of the population. How do we encourage EAs to instead produce individuals possessing *all* of these skills?

One approach might be to use Novelty Search, which disregards the traditional fitness-based objective in lieu of producing *novel* individuals [19]. By encouraging agents to express different behaviors from other individuals, Novelty Search overcomes issues of deception [19] and has been shown to produce agents with general exploratory skills [9]. At a cursory glance, one might therefore expect Novelty Search to produce generalists. However, we argue that upon further reflection, it should be clear that Novelty Search actually produces specialists, in which each individual possesses a subset of skills, but no one organism possesses all skills.

**Fig 1:**
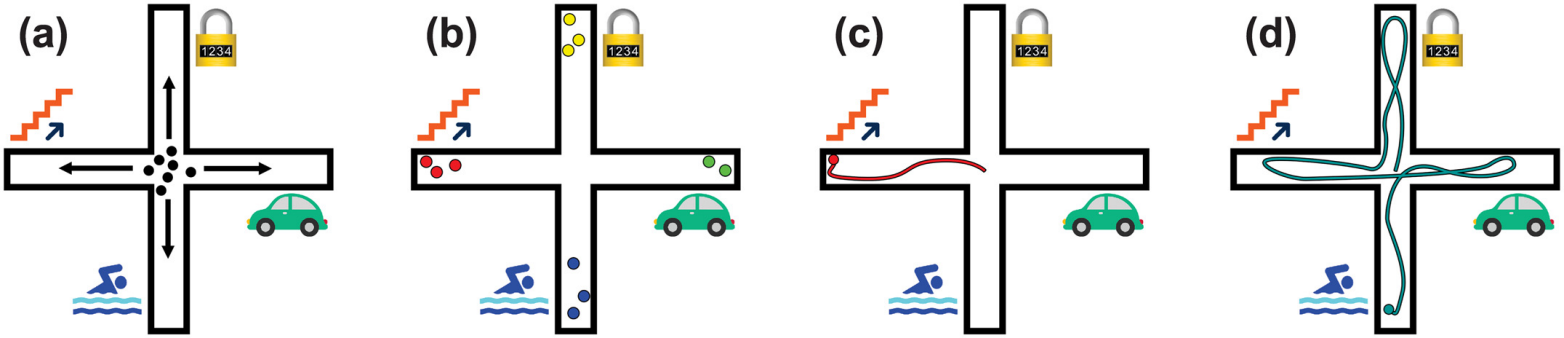
We hypothesize that agents produced by Novelty Search will specialize in a multiple-skill domain. In this example domain, agents must climb stairs to move west, swim to move south, pick locks to move north, etc. **(a)** At the beginning of evolution, individuals placed in the center of this maze are unlikely to move very far, creating a pressure to explore outwards as the “available novelty” in the center of the maze is quickly exhausted. **(b)** Sub-populations of Novelty Search explore outwards, resulting in some individuals that can climb stairs and others that can swim; however, **(c)** the algorithm is unlikely to produce individuals that possess *all* of these skills, resulting in specialists. **(d)** We hypothesize that an agent produced by Curiosity Search will instead be rewarded for exploring all of its domain and acquiring many skills.

To illustrate this, imagine a maze environment in which each branch of the maze requires wholly different skills to explore (Fig 1, further discussed in Sec. Novelty Search). The population produced by Novelty Search may explore everywhere, but *individuals* have no incentive to explore their entire environment so long as they are sufficiently different from other individuals. We might thus expect Novelty Search to produce sub-populations of specialists, each exploring only one branch of the maze. However, we ultimately want to produce agents that explore *all* branches of the maze and acquire *all* the skills necessary to do so.

Here we propose a new algorithm called *Curiosity Search*, which aims to produce individuals that explore their entire environment and acquire as many skills as possible within their lifetime. Like Novelty Search, Curiosity Search operates without a traditional fitness goal [19], instead pressuring agents to express as much *intra-life* novelty as possible. Unlike Novelty Search, this novelty is measured not against the population, but against the individual’s own accomplishments. In other words, if an agent learns how to swim during its lifetime, that behavior is considered novel—regardless of whether the rest of the population has already acquired that skill. Further, each agent is pressured to acquire additional skills (e.g. stair-climbing) while retaining its existing skill set.

We begin by describing our ultimate vision of Curiosity Search, which pressures individuals to “do something new” during their lifetime, similar to the biological mechanisms of curiosity we observe in natural animals. Because this vision presents a large number of technical hurdles, we test a simpler version of Curiosity Search within the domain of maze navigation. We show that Curiosity Search produces generalists in this domain; further, that a traditional implementation of Novelty Search produces specialists. We reveal that Novelty Search can be modified to produce individuals very similar to those of Curiosity Search—although the agents produced by Curiosity Search retain a significant exploration edge. Finally, we show that Curiosity Search is a useful helper objective when combined with Novelty Search, producing generalists that acquire significantly more skills than either treatment alone.

## Novelty Search

Traditional EAs are often susceptible to deception, in which their greedy nature causes them to become stuck at sub-optimal solutions known as local optima [20]. Novelty Search helps avoid deception by instead encouraging individuals to be behaviorally different from previously produced individuals [19]. Agents are compared by their *behavioral distance* from the rest of the population and also from an archive, which contains a history of prior individuals to prevent backtracking. The distance function can take different forms; for example, in a maze environment, one possible metric is the distance between the final locations of each agent, thereby rewarding individuals for ending up as far away from each other as possible.

Novelty Search pressures individuals to be behaviorally different from one another; at least initially, we might expect that those individuals will express a wide range of behaviors and thus be generalists. This hypothesis might seem even more compelling given that Novelty Search was recently shown to reward the development of general skills in the organisms it produces [9]. However, that work did not examine whether Novelty Search produces individuals that acquire all or most of the skills in their environment (and the data in that paper suggests otherwise) [9].

Upon further reflection, however, it is clear that by only comparing individuals to the rest of the population, Novelty Search will produce specialists. To illustrate this point, suppose individuals are placed in the center of a maze in which progress requires the acquisition of distinct skills (Fig 1). At the onset of evolution, organisms will probably possess little or no skills; because they do not move very far, available novelty in the maze center will rapidly diminish, creating an outward pressure to explore. In response, individuals will begin to explore along a single branch and acquire a subset of skills. If a portion of the population explores in a single direction (e.g. East), there is an incentive for other subsets of the population to explore in a different direction (e.g. West). Thus, while the population itself might extend everywhere, no one *individual* would acquire all of the skills in this domain. In other words, a multitude of behaviors might be rewarded (e.g. swimming, driving, climbing stairs), but an individual only has to possess *one* of these skill sets to be novel—there is no incentive to acquire all of the skills.

One might argue that this specialization is simply a consequence of comparing individuals by their final location, and that we could prevent this problem by using a different notion of behavioral distance. On problems other than maze navigation (e.g. a biped gait task [19]), Novelty Search has indeed been implemented with a behavioral distance that rewards intra-life diversity.

We agree that an intra-life behavioral distance function should alleviate specialization (and test this possibility), but argue that the same fundamental issue will persist regardless of the function used. At best, Novelty Search might be constructed with a behavioral distance function based on the number of skills performed by an agent during its life. This function would reward the production of at least one individual that performs all skills because the first occurrence of that behavior would be considered novel. However, Novelty Search would also reward individuals for acquiring no skills, only one skill, etc. Because the majority of the search would not be directed towards producing generalists, it seems unlikely that Novelty Search would accomplish that goal (at least, within a reasonable amount of time). However, whether the production of generalists is best served by a direct pressure (via Curiosity Search) or a less-direct approach (via Novelty Search with the aforementioned distance function) remains an empirical question, and serves as a fundamental motivation for our experiments.

## Curiosity Search

Curiosity Search is designed to produce generalists by promoting *intra-life* novelty within each individual, tasking organisms with the directive: “do something new.” This algorithm pressures individuals to express as many different behaviors as possible during their lifetime, instead of rewarding for improved performance on only one or a few behaviors as is common in objective-based searches. Curiosity Search has two main components: an *intra-life novelty score* (Sec. Intra-Life Novelty Score) that quantifies the types of behaviors rewarded by the algorithm; and a *fitness function* (Sec. Fitness Function) rewarding individuals for expressing as many unique behaviors as possible. Optionally, agents can be given an *intra-life novelty compass* (Sec. Intra-Life Novelty Compass), which provides a guide as to which actions and behaviors might be considered novel.

Previous work has suggested that encouraging novel behaviors within an individual’s lifetime can result in agents that explore and improve their understanding of the environment. Machine learning models of intrinsic motivation, which attempt to imitate the curiosity seen in animals, have resulted in agents that can successfully interact with objects in a playpen environment [7], autonomously identify and pursue goals to maximize their understanding of the world [21], and even learn tactile skills [22]. However, these models cannot be directly applied to an evolutionary domain in which there is no learning; furthermore, they do not shed insight as to how intrinsic motivation might have evolved in the first place. One work showed that an evolutionary process can encourage intra-life exploration via a system of diminishing rewards that punishes agents for repeating the same high-scoring (but not necessarily optimal) behaviors [23]. However, in that work agents were only required to explore to avoid exploiting one or a few behaviors; there was no explicit pressure for agents to do *everything* as in Curiosity Search.

Another work adopted a similar approach to that proposed in this paper, pressuring agents to express high entropy in the *sensorimotor states* they visited [24]. Although that work did not specifically investigate the problem of producing generalists, it did provide a theoretical framework for promoting exploration without any domain knowledge and was later proven to be effective at promoting exploration on a maze navigation task [25]. It may be worth studying whether their approach also produces generalists or whether Curiosity Search might be made more general by adopting their methodology. A similarly inspired, but not directly related approach is the maximization of agent *empowerment* [26], in which agents are tasked with increasing their ability to influence the world around them. While empowerment is quite different from the pressure to “do something new,” both principles assume long-term evolutionary benefits from increasing an agent’s immediate ability to understand and explore its domain, as opposed to maximizing traditional fitness goals.

The ultimate goal of Curiosity Search is to instill agents with a perpetual drive to explore their environment and acquire new skills. We hypothesize that the combination of Curiosity Search with an Innovation Engine (which has the potential to automatically recognize new and interesting behaviors [27], but does not reward the acquisition of multiple skills) might create an open-ended process in which agents express new and increasingly complex behaviors without any human-specified goals. However, Innovation Engines are not yet capable of identifying interestingly new behaviors in high-dimensional, unlabeled sensor-action spaces (such as the continuous input/output streams of a robot) [27]; construction of this longer-term vision of Curiosity Search is not yet feasible. We thus show the merits of rewarding intra-life diversity via a simpler version of Curiosity Search in which the set of rewarded behaviors has been manually determined.

We call this algorithm “Curiosity Search” because the reward scheme encourages evolution to search for curious agents. Our approach to encouraging curiosity is different from previous work (e.g. by Schmidhuber [18] and Oudeyer [7]) in that it produces curiosity simply by rewarding agents for doing something they have not yet done within their lifetime. In this initial paper on Curiosity Search, there is no intra-life reward provided for performing a new action; likewise, skills in this work are acquired across evolutionary time and hardwired into each agent’s genome, and agents do not have the ability to learn *within* their lifetime. However, we do provide an intra-life novelty compass that tells the agent in which direction it should head to explore a new area. Since evolution is encouraged to search for agents that head in the direction indicated by this compass, agents can be seen as implementing an abstraction of curiosity because they constantly attempt to explore new areas. An interesting question for future work is whether providing Curiosity Search with more sophisticated cognitive capabilities (e.g. agent memory, intra-life plasticity, reinforcement learning, etc.) will enable Curiosity Search to produce agents with more sophisticated and realistic analogues of biological curiosity.

### Intra-Life Novelty Score

The intra-life novelty score determines how many unique behaviors have been expressed within an individual’s lifetime. This score can be implemented in several ways: on simpler problems, such as the maze exploration task used in this paper, one might create a discretization in which there are *K* possible behaviors and the score is the number of behaviors in *K* that have been expressed. On more complex problems, the intra-life novelty score might instead demarcate behaviors using a distance function (e.g. there are many different types of gaits; one possible score might use the differences in speed, actuator usage, etc. to decide when a new type of gait has been expressed).

The choice of this scoring function will have a substantial effect on the type of individuals produced. A score reliant on reaching environmental landmarks (e.g. rooms of a house) will encourage novelty in the physical locations visited by an individual. A score that demarcates between different types of actuator usage on a robot gait task would instead encourage individuals to express many different forms of locomotion within their lifetime [28]. Regardless of its implementation, this scoring function returns a number (in this paper, a non-negative integer) indicating how many unique behaviors have been expressed during an individual’s lifetime.

### Fitness Function

In its simplest form, the fitness function might be synonymous with the value given by the intra-life novelty score (as is done in this work). However, this function can also be extended to accommodate domain-specific mechanics (e.g. punishment for being caught by a predator). As long as the fitness function pressures individuals to perform as many different behaviors as possible, Curiosity Search does not otherwise limit the addition of any other fitness constraints.

One alternate implementation of Curiosity Search might reward agents for their performance on all skills (e.g. via a fitness function *f* = *a* × *b* × … × *z*, where each *a*, *b*, …, *z* represents the performance on each skill). This version of Curiosity Search emphasizes not just acquiring many skills, but *doing well* on all of them. However, to be in the spirit of Curiosity Search, the number of skills should be quite high and many of them might not be things that the user is ultimately interested in having the robot perform. Instead, the intent is that many of these skills will serve as *stepping stones* to other skills that are of more interest to the user (a technique shown to be useful in other domains [27]).

In this paper, fitness in Curiosity Search only considers the number of unique behaviors expressed. An agent that learns how to swim and drive has expressed the same number of behaviors as an agent that instead learns how to climb stairs and pick locks; therefore, both have the same fitness. Further, the order of behaviors is unimportant; it does not matter whether an agent first learns to drive and then swim, or swim and then drive. Consequently, a single fitness score in Curiosity Search might conflate a plethora of diverse agents. Although there is no explicit pressure for diversity, this fitness conflation might nonetheless produce a diversity of solutions. Because population diversity is known to help avoid local optima [29], Curiosity Search might be less prone to deception than other traditional objective-based searches. However, as discussed below, Curiosity Search does benefit from the inclusion of a diversity-promoting helper objective.

### Intra-Life Novelty Compass

Agents in Curiosity Search can be given an intra-life novelty compass, which indicates to the agent which behaviors would be considered novel within the environment. Although this mechanism is not essential to Curiosity Search, its addition may help agents make better decisions about how to explore their domain and may thus help bootstrap/accelerate the search for novelty.

In its most general implementation, the compass informs an agent which types of behaviors would be rewarded by the intra-life novelty score if they were performed. This is easily implemented if an agent is to be rewarded for physically reaching new areas; the compass can simply point towards the nearest unexplored room or region. Additional research is required to determine how to provide such information in general. However, one possible approach would be to identify and direct the agent towards areas of *high expected learning* [7, 18], because new knowledge often leads to the ability to perform new skills.

## Methods

### Problem Domain

**Fig 2:**
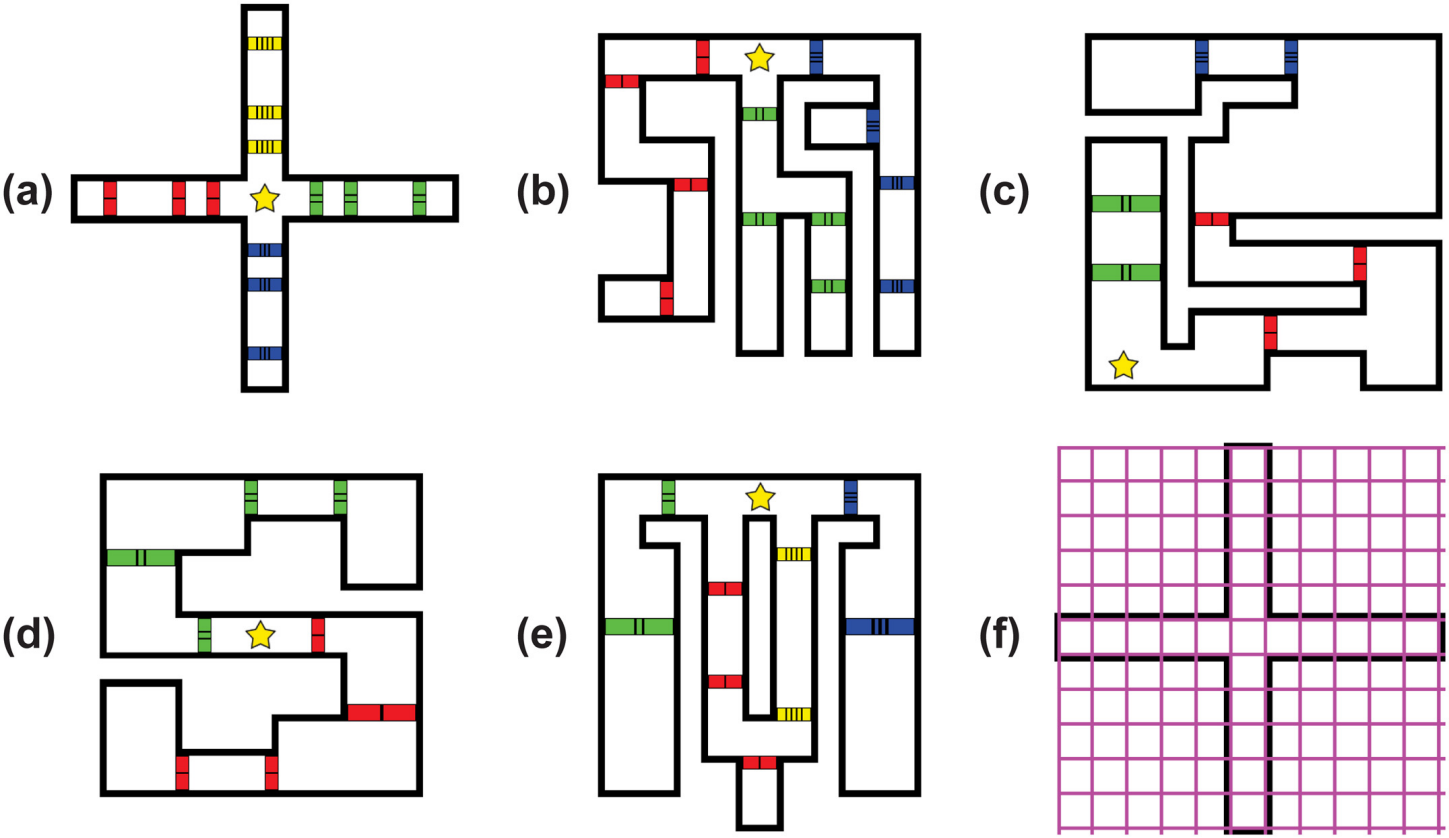
Maze Environments and Discretization. Agents are tasked with navigating either the **(a)** Cross Maze, **(b)** Finger Maze, **(c)** Hard Maze, **(d)** S Maze, or **(e)** Tri Maze. Each maze contains red, blue, green, and/or yellow doors and is discretized via the grid shown in **(f)**. The star represents the start position; there is no explicit goal.

We adopt the classic domain from Novelty Search, in which wheeled robots are evolved in a 2D maze [19]. Specifically, we evolve robots in one of five mazes (Fig 2), each of which has a single start position, no explicit goal, and a number of multicolored doors. We chose a variety of environments to ensure the generality of our results.

Robots are controlled by feedforward artificial neural networks (ANNs) with connection weights within the range [-1, 1] and a sigmoid activation function at each neuron. Each robot has 6 directional range-finders for detecting walls, as in previous work [19]. Robots also have a separate door-opening neuron for each type of door (see Sec. Neural Network Structure). Because each of these neurons must be independently wired, each type of door can be thought of as representing a functionally different skill. Doors are impassable until opened by a robot; after opening, a door remains open for the duration of the simulation.

Mazes are discretized as in Fig 2f. We tested several different combinations of door colors (e.g. in the Cross Maze, substituting the three red doors of the west branch with a red door, a green door, and a blue door, etc.); all produced similar results. Simulations in the Cross Maze last 2,500 time steps; in all other mazes, simulations last 5,000 time steps due to their larger navigable area. We conduct simulations via a modified version of the Fastsim simulator [30].

Curiosity Search’s mandate “do something new” can be measured in many ways; we translate that directive into *“go somewhere new”* because the latter is easy to quantify. In this domain the translation works especially well because exploration requires the acquisition of distinct skills (i.e. opening different types of doors). However, the directive “go somewhere new” may also be useful in a more general context. For example, imagine tasking a robot with going somewhere new in a simulated or real house: the robot would have to learn how to open doors, navigate hallways, climb/descend stairs, etc. to reach new areas. In video games, going somewhere new might entail solving puzzles and avoiding or killing enemies. Thus, this simple method of implementing Curiosity Search might serve as powerful general reward scheme for encouraging agents to acquire a wide and diverse skill set.

When describing our experiments in this work, we treat skill acquisition and exploration as interchangeable terms because they are highly correlated (i.e. it is impossible to explore the maze without also opening doors) and because the skills themselves are simple. However, we note that equating these two concepts may not always be appropriate, especially in domains for which the set of obtainable skills is not necessarily tied to “going somewhere new” (e.g. a robot might learn many different gaits without ever leaving the room in which it is initially placed).

For an intra-life novelty compass, robots are given six pie-slice tile sensors, which return the number of unvisited maze tiles, and corresponding “newness,” in any given direction. Robots also possess six pie-slice sensors for each door type, which return the Boolean value 1 if an unopened door is detected in a given direction and 0 otherwise. All sensor distances are unbounded, but robots cannot see through walls or doors.

We hypothesize that in this domain (1) Novelty Search will produce specialists, each of which only explores one branch of the maze, and (2) Curiosity Search will instead produce generalists that explore significantly more of their environment (Fig 1). This domain attempts to abstract environments wherein different skills are required to explore the environment. Although rewarding intra-life behavioral novelty is a general principle that should apply to many domains, it remains a challenge how to recognize and reward new types of behaviors that will lead to the acquisition of complex skills. For example, learning how to pick a lock via Curiosity Search would require rewarding the performance of a variety of subtly different movements in different orders and combinations. However, doing so may also prevent the acquisition of skills that require vastly different types of motion (e.g. cartwheels) if the search spends most of its time generating subtly different movements that are not helpful for learning things like cartwheels. Thus, while the general idea behind Curiosity Search—encouraging the performance of new behaviors within a lifetime—is a promising, general paradigm for encouraging skill acquisition and the production of generalists, much work remains to be done regarding how best to automatically and effectively recognize new types of behaviors that will lead to the acquisition of a diversity of complex skills.

### Neural Network Structure

**Fig 3:**
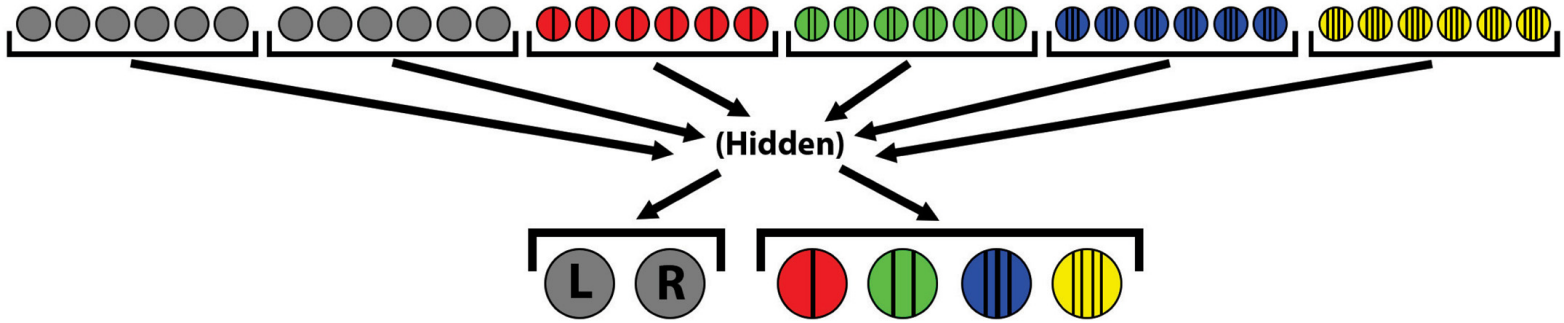
Base ANN structure for all experiments. On top are inputs for the range-finders, tile sensors, and the red, green, blue, and yellow door sensors, respectively. Signals propagate via a hidden layer to the outputs: the left/right motors and the red, green, blue, and yellow door-opening mechanisms.

Robots are driven by the ANN described in Fig 3. As in previous work [19], two neural outputs constrained between the values [-3, 3] drive the robot’s left and right motors. Robots possess *K* additional output neurons, one for each type of door (in our experiments, *K* = 4). Doors can be opened only if the robot is within range of that door’s center (twice the door’s width).

To determine if a door should be opened, a softmax is applied at every simulation step to all door output neurons (a vector **N**) to produce a new vector **N**^**′**^ = *softmax*(**N**). If the maximum output *M* = *max*(**N**^**′**^) is *c* times greater than any other output in **N**^**′**^, where *c* (here, 2.0) is the *confidence threshold*, the door is considered opened. This function requires ANNs to be reasonably certain not only that a door is red, but also that the door *is not* green, blue, or yellow. We also tried an implementation in which all other outputs except for the candidate door must be zero; however, this required more time for the ANNs to learn and did not otherwise impact our results.

We considered adding a punishment for giving the wrong answer (e.g. activating the red door neuron when a yellow door is present). However, all tested forms of punishment severely reduced individual exploration in all treatments. We note that any punishment—whether by “freezing” a robot in place or by lowering its final fitness—may actually *discourage* exploration because it punishes the natural process of trial-and-error. Thus we do not use any punishment model in our experiments.

### Controls and Parameters

We test four treatments: **Curiosity Search**, **Novelty Search**, **Curiosity Search + Novelty Search** combined as two objectives in multi-objective optimization, and a baseline algorithm **Target Endpoints** (based on MAP-Elites [31]). For Curiosity Search and Novelty Search we use the rtNEAT software package, which evolves networks via the NeuroEvolution of Augmenting Topologies (NEAT) [32]. Because rtNEAT does not support MAP-Elites or multi-objective optimization algorithms, we use the Sferes evolutionary framework [30, 33] for Target Endpoints and the multi-objective combination of Curiosity Search and Novelty Search. The simulator and neural network setup in both software packages is identical. Sferes does not contain an implementation of NEAT, but it does evolve networks via a direct encoding of neural networks that is inspired by NEAT; we found results from Sferes to be qualitatively similar to those produced by rtNEAT. All experiments have a population size of 125 and last 2,000 generations; 25 runs were given for each experiment. We deviated from the traditional population size of 250 to enable a more thorough investigation given our limited computational resources and because preliminary experiments found no qualitative difference between a population size of 250 and 125. Statistical significance scores are computed via a two-tailed Mann-Whitney U test (the preferred test for EAs, which do not produce normally distributed results).

**Curiosity Search** (*CS*) uses the fitness function *f* = *t* + *d*, where *t* is the number of *unique* grid tiles touched during simulation and *d* is the total number of doors opened. In other words, both the exploration of a new grid tile and the opening of any door are considered unique behaviors that should be rewarded by the algorithm. We also ran a control in which the fitness function was *f* = *t* (tiles only) to determine whether agents could acquire door-opening skills without any direct pressure (or reward) for opening them. Because the results using fitness function *f* = *t* were qualitatively unchanged as compared to *f* = *t* + *d*, we use the latter function in this work, which rewards both for exploring the domain and opening doors. A comparison of each fitness function is provided in S4 Fig.

Because Curiosity Search optimizes a single objective, it could suffer from *deception* [19, 20] and might benefit from added diversity, like many other objective-driven EAs [29]. We combine **Curiosity Search + Novelty Search** using NSGA-II [34], adopting default NSGA-II parameters provided by Sferes. As described in Novelty Search (below), we test two behavioral distance functions: endpoints (*CN*_*E*_) and trajectories (*CN*_*T*_).

We investigate two different behavioral distance functions for **Novelty Search**: (1) the traditional Novelty Search distance function for this domain (*NS*_*E*_), which compares individuals via their endpoints and which we believe will produce specialists, and (2) a behavioral distance from Lehman and Stanley 2011 [19] that instead compares the entire trajectory of each individual (*NS*_*T*_). The latter metric aims to make Novelty Search more *lifetime-aware*; in considering more than just the final position, we expect that Novelty Search will produce at least a few generalists and compare more favorably to Curiosity Search. Following preexisting work [19], distance is computed by the averaged Manhattan distance between each coordinate (i.e. ||**a** − **b**||_1_ ÷ *k* where *k* is the length of vectors **a** and **b**; this has sometimes been mistakenly referred to as Euclidean distance). Both metrics use the same parameters as Velez and Clune 2014 [9]. For *NS*_*T*_, we chose trajectory-based Novelty Search over a tile-based implementation to enable comparisons to the former, which has already been shown to work well on other tasks [19] and for which a tested implementation was already provided in rtNEAT and Sferes.

It might not be apparent why novelty in trajectories is comparable to the “tiles explored” of Curiosity Search. This relation can be intuitively understood by contrasting trajectories with the example of specialization in Fig 1. In an endpoints-version of Novelty Search, specialization occurs because if e.g. the west branch is most novel, there is no incentive for an individual to first head east before fully exploring the west branch—only the final location matters. However, a trajectory-version of Novelty Search *does* reward an individual for this behavior (provided other individuals are not doing the same), because the resulting variation in trajectory makes that individual more novel.

However, this does not mean that Novelty Search in trajectories is identical to Curiosity Search. Curiosity Search rewards individuals for doing *everything* (or, in our simplified version, for going *everywhere*); Novelty Search instead rewards individuals for being *different* from other individuals. With a lifetime-aware behavioral distance metric (e.g. trajectories), a population in which each individual is rewarded for being different would reward an individual that does everything, but would also reward individuals that do nothing, only do one thing, etc.

**Target Endpoints** (*TE*) is a new algorithm that rewards individuals for minimizing the distance between their final position and any of several specific points in the maze. *TE* uses the default version of MAP-Elites [2, 31] provided by Sferes. The behavioral bins comprise a one-dimensional vector of locations **L** in the maze domain, in which each location represents a grid tile of the maze discretization. Performance for each location in **L** is measured as the final distance of the organism from the center of that grid tile. If an individual outperforms the current champion for any *l* ∈ **L**, it replaces that champion.

All of our mazes require returning to the start position at least once in order to explore the entire domain (Fig 2). However, individuals evolved under *TE* only need to reach a target point and then stop; there is no reward for agents to explore anywhere else along the way. Further, even a partial return to the maze start is unlikely to be rewarded—other individuals have likely already become champions of the intermediate tiles (and have likely already achieved near-perfect fitness, making them difficult to replace). We expect *TE* is therefore predisposed towards specialization, making it a useful empirical lower-bound for what an extreme version of specialization looks like.

## Results and Discussion

### Novelty Search Specialization

**Fig 4:**
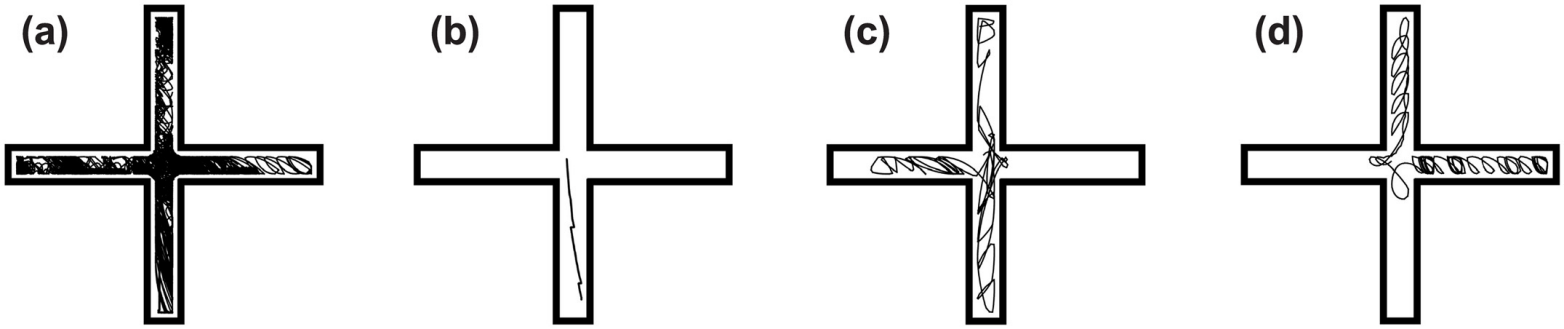
Novelty Search produces specialists and Curiosity Search produces generalists. **(a)** Fifty trajectories from a *NS*_*E*_ population are overlaid to show the population-level coverage (results typical for all mazes; see S1 Fig). Novelty Search explores the entire domain, but **(b)** a typical *NS*_*E*_ individual only explores a small portion of that domain, whereas **(c)** a typical *CS* individual tends to explore significantly more. **(d)** *NS*_*T*_ instead produces individuals that explore similarly to, although not usually as much as, Curiosity Search.

**Fig 5:**
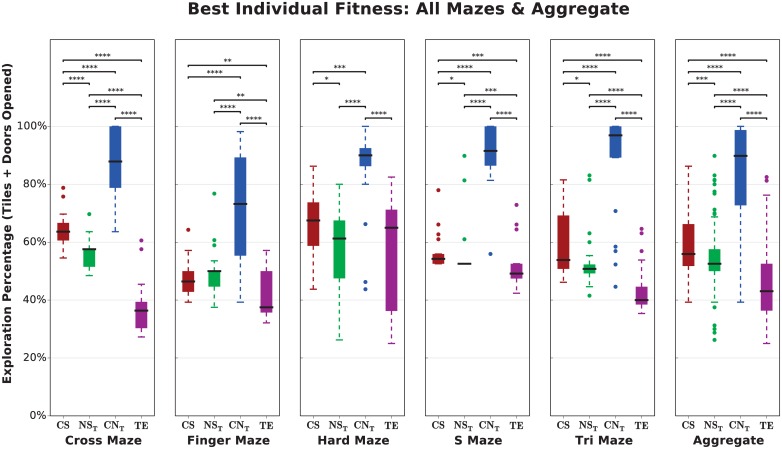
Across almost every maze, Curiosity Search outperforms Novelty Search in producing generalists. Aggregate performance is obtained by pooling each treatment’s results across every maze. Even when agents produced by Curiosity Search do not explore more than those of Novelty Search (e.g. on the Finger Maze), their exploration still matches that of Novelty Search (*p* ≥ 0.05).

We manually examined over 500 randomly-chosen *CS*, *NS*_*E*_, and *NS*_*T*_ trajectories; the most common results are shown in Fig 4 and S1 Fig. Although a *population* in the endpoints version of Novelty Search explores everywhere (Fig 4a), a typical *individual* only explores a single branch (Fig 4b). In contrast, Curiosity Search produces generalists that explore a large portion of their domain and acquire many skills (Fig 4c). Organisms produced by *NS*_*T*_ behave more similarly to those of *CS* (Fig 4d), but do not explore quite as much of their environment as Curiosity Search (Fig 5, *NS*_*T*_ vs. *CS*).

**Fig 6:**
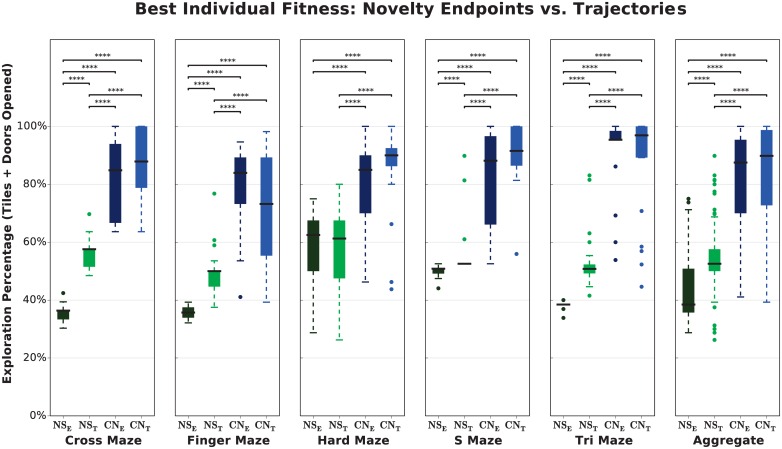
A lifetime-aware evaluation mechanism appears valuable for producing generalists, regardless of the algorithm used. The best individuals in trajectory-based Novelty Search (*NS*_*T*_) explore more than those of endpoint-based Novelty Search (*NS*_*E*_). The difference between trajectories and endpoints is not significant when Curiosity Search is added as a helper objective (*CN*_*E*_ vs. *CN*_*T*_).

As expected, switching to a behavioral characterization that is more lifetime-aware increases exploration. On four out of five mazes, the best-exploring individuals of *NS*_*E*_ explore less than 50% of the domain, while those of *NS*_*T*_ explore significantly more (Fig 6, *NS*_*E*_ vs. *NS*_*T*_, *p* < 0.0001). The combination of Curiosity Search and Novelty Search in multi-objective optimization increases exploration by 20-50% and causes the difference between endpoints and trajectories to completely disappear (Fig 6, *CN*_*E*_ vs. *CN*_*T*_, *p* ≥ 0.05), possibly because Curiosity Search already provides the intra-life pressure requisite to explore this domain.

From this data, we conclude that the presence of any lifetime-aware evaluation mechanism may be valuable, regardless of the algorithm used, wherever generalists are desired. Because trajectories in Novelty Search offer better exploration on this task and will thus make a fairer comparison to Curiosity Search, we exclusively use *NS*_*T*_ and *CN*_*T*_ for the rest of our results.

### Maze Exploration

**Fig 7:**
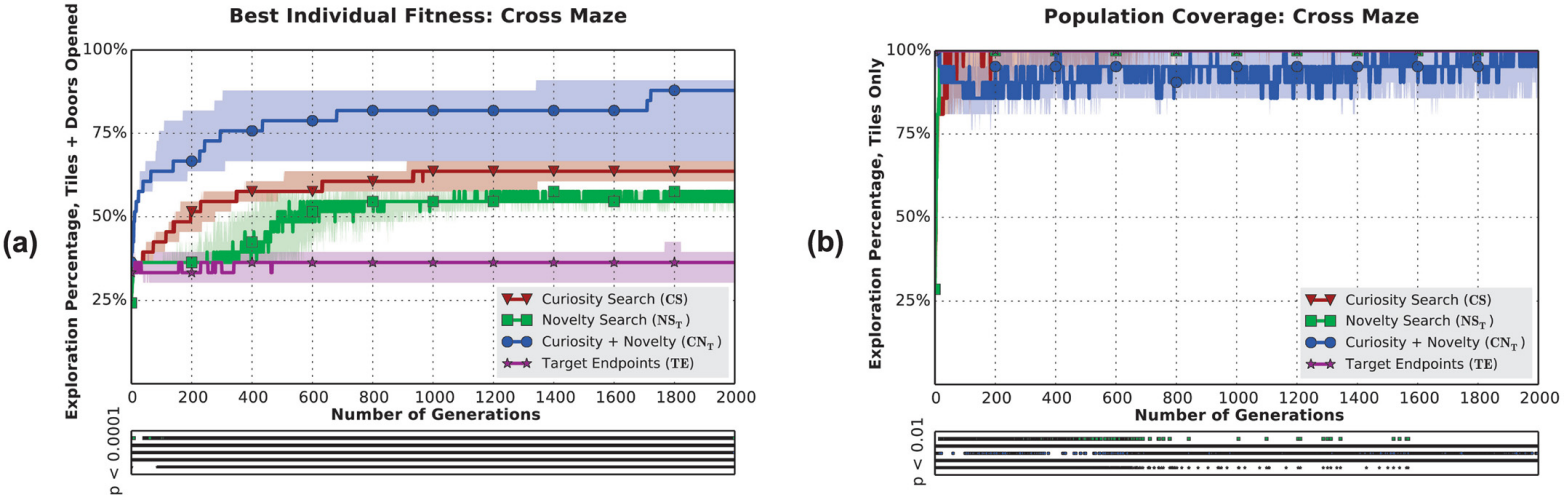
Curiosity Search produces individuals with greater exploratory skills than Novelty Search, but the *populations* of all treatments explore the entire maze. Results show the Cross Maze only (data from other mazes shown in S2 Fig). **(a)** Both curiosity treatments significantly outperform *TE* and *NS*_*T*_ over evolutionary time. *CN*_*T*_ produces individuals that explore more than either algorithm alone, suggesting that Curiosity Search may be a good helper objective for producing generalists. **(b)** The population of every treatment, even those that produce specialists (such as *TE*), can explore the entire maze, revealing that population diversity alone is not sufficient to encourage generalists.

In the Cross Maze, both curiosity treatments (*CS*, *CN*_*T*_) significantly outperform trajectory-based Novelty Search in producing individuals that explore their domain over the entirety of evolution (Fig 7, *p* < 0.0001). Individuals produced by combining Curiosity Search and Novelty Search (*CN*_*T*_) explore almost 90% of the maze, far more (approximately 15-25%, *p* < 0.0001) than either treatment alone, indicating that Curiosity Search (1) benefits from the inclusion of a diversity-promoting objective and (2) is possibly prone to deception in this problem. Results for other mazes are similar (see S2 Fig).

Individuals produced by Target Endpoints explore only a single branch of the Cross Maze, confirming our expectation that the algorithm would produce specialists. *NS*_*E*_ achieved a nearly identical end-of-evolution performance as *TE* (just under 40%, Fig 6 cross-referenced with Fig 5), providing further evidence that Novelty Search produces specialists.

These trends are consistent across all mazes at the end of evolution (Fig 5). *TE* explores significantly less than every other treatment (*p* < 0.01; in aggregate, *p* < 0.0001 and exploration is never greater than 50%). Agents produced by Curiosity Search either match those of Novelty Search (*p* ≥ 0.05) or significantly outperform it (*p* < 0.05; in aggregate *p* < 0.001). Lastly, and perhaps of even more interest, *CN*_*T*_ outperforms all other treatments (*p* < 0.001), producing individuals that explore between 75-98% of their domain—almost the entirety of every maze.

### Do Doors Represent Distinct Skills?

One could argue that the doors in our domain might not constitute distinct, non-trivial skills because agents are not prevented from cycling through door outputs and brute-forcing their way through the maze. There is nothing directly preventing evolution from producing a “dumb” agent that bounces off of walls and opens doors quite by accident.

**Fig 8:**
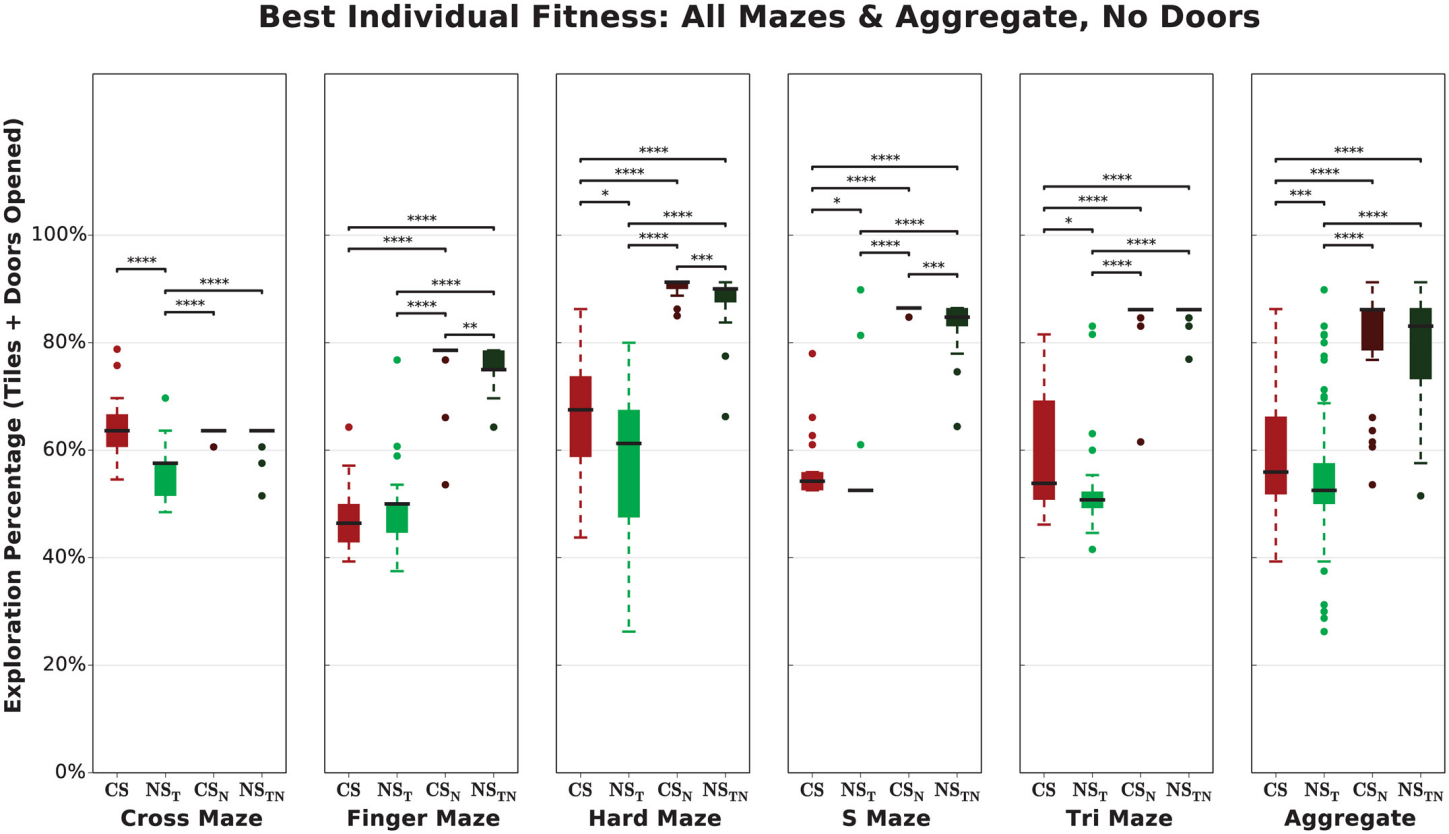
Removing doors increases exploration and causes the difference between Curiosity Search and Novelty Search to disappear. Treatments in which doors were removed are subscripted with *N* (*CS*_*N*_, *NS*_*TN*_); all other treatments contain doors. If the doors were merely obstacles requiring no skill to open, we would expect little or no change in either algorithm’s ability to explore the domain after doors are removed. However, exploration greatly increases when doors are removed and the difference between treatments disappears almost entirely, suggesting that the doors in these experiments are an abstraction of distinct skills, which *CS* seems better able to acquire.

To ascertain whether this phenomenon occurs, we test *CS* and *NS*_*T*_ in each maze with and without doors. If the doors are merely obstacles requiring no skill to open, removing them should have little to no effect on our results: *CS* should still slightly, but significantly, outperform *NS*_*T*_ and overall exploration percentages should remain similar. However, we found that both *CS* and *NS*_*T*_ agents explore nearly twice as much of their environment when the doors are removed (Fig 8, *p* < 0.0001 except for the Cross Maze, in which there is no significant change). Furthermore, the exploration gap between Curiosity Search and Novelty Search disappears almost entirely; in aggregate there is no difference (*p* ≥ 0.05) after the doors are removed.

These results suggest that opening each type of door does in fact represent a distinct and non-trivial skill (at least for traditional evolutionary algorithms). Additionally, evolution does not appear to produce a successful version of the hypothesized “dumb” agent. Lastly, Curiosity Search is better able to acquire these skills than Novelty Search in this domain.

An additional way to test whether the doors in these experiments represent distinct skills is to directly measure an evolved agent’s ability to open each type of door. If the different doors don’t represent different skills, an agent should be able to open all of them with equal ability. We tested this idea by placing agents in an environment in which the only task is to open doors; agents were graded on how many *different types* of doors they could open (red, green, blue, etc.). We found that Curiosity Search agents open more types of doors than those of Novelty Search, reinforcing our claim that doors are non-trivial skills that Curiosity Search is better able to acquire (see S3 Fig for the experimental details and results).

### Transfer Experiments

One might ask whether the high-exploring individuals produced by Curiosity Search have acquired *general* skills or have simply memorized a trajectory for a single domain. Previous work has demonstrated that Novelty Search produces agents with general exploratory skills [9]. We test whether *CS* produces agents with similar or better exploration skills than Novelty Search by (1) evolving individuals in the Cross Maze, (2) transferring the best-exploring individuals into the other four mazes, and (3) evaluating their ability to explore the new environment, both without and with further evolution.

#### Transfer Without Further Evolution

**Fig 9:**
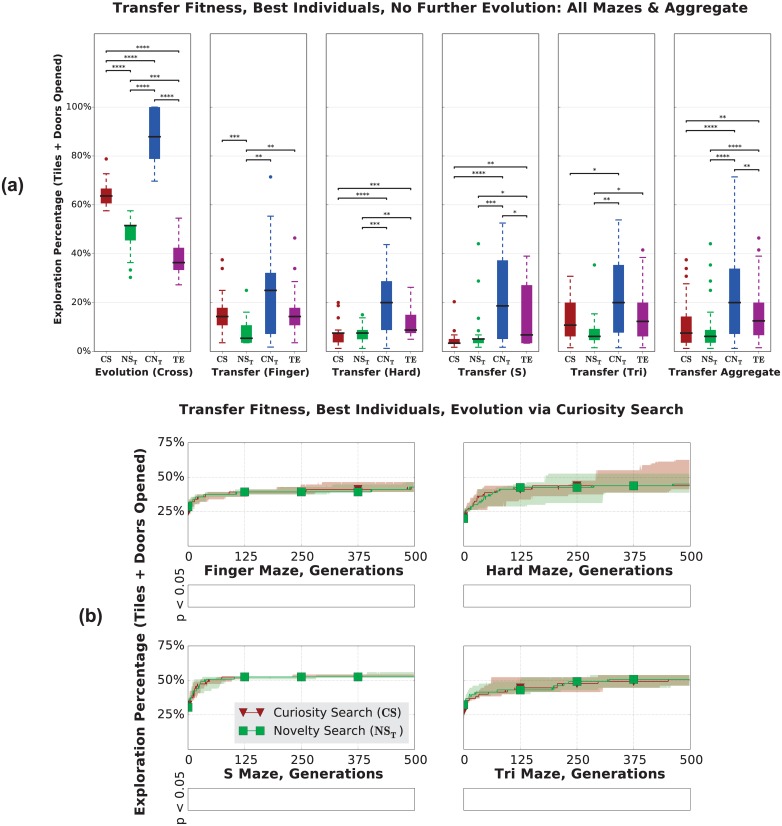
Curiosity Search agents acquire general exploration skills in a manner similar to Novelty Search. **(a)** Exploration after individuals are evolved in the Cross Maze, transferred to the other mazes, and evaluated without any further evolution. *CS* transfers at least as well as *NS*_*T*_, but only rarely explores more in the new domain. Surprisingly, Target Endpoints, which explored the least in the original maze, outperforms all treatments after transfer except for *CN*_*T*_. **(b)** After further evolution, there is no difference between *CS* and *NS*_*T*_, suggesting that both may equally evolvable.

Without any further evolution, we found that Curiosity Search matches, but only rarely exceeds, the transfer performance of *NS*_*T*_ (Fig 9a); in aggregate there is no significant difference between the two (*p* ≥ 0.05). However, the combination of Curiosity Search and Novelty Search (*CN*_*T*_) explores 10% more than any other treatment (*p* < 0.01 in aggregate), again making it superior to either algorithm alone. Curiosity Search seems to have acquired general exploration skills in a similar manner to Novelty Search [9]. Agents produced by both Curiosity Search and Novelty Search have not simply memorized a high-performing trajectory; they have instead acquired a general ability to explore their environment.

Interestingly, while Target Endpoints has consistently explored less than all other treatments, it performs very well after transfer, exploring more than *CS* and *NS*_*T*_. We cannot offer a definitive explanation for this occurrence. However, we suspect that because most of the agents produced by *TE* in the Cross Maze develop remarkably straight trajectories, and because most of our mazes have straight corridors near their starting positions (Fig 2), *TE* may be better suited to transfer to these mazes.

#### Transfer With Further Evolution

Given that agents produced by Curiosity Search transfer at least as well as Novelty Search, it is natural to ask whether the general skills they acquire are of any benefit to further evolution. We take the individuals produced by *CS* and *NS*_*T*_ and evolve them via a common algorithm (Curiosity Search) in the new maze. Code incompatibilities prevented us from transferring *CN*_*T*_ and *TE* organisms from Sferes to rtNEAT, where Curiosity Search is implemented.

There was no significant difference (*p* ≥ 0.05) between *NS*_*T*_ and *CS* after further evolution (Fig 9b). Also, the original exploration difference disappears immediately at the onset of evolution, suggesting that the individuals produced by *CS* and *NS*_*T*_ are equally evolvable under Curiosity Search.

## Future Work

The evidence presented in this paper supports the claim that Curiosity Search produces generalists. However, we wish to further investigate that claim by testing Curiosity Search in different environments, such as the traditional biped experiment [19] or the more complex navigation and enemy-avoidance tasks seen in video games [35]. It would also be interesting to apply Curiosity Search to machine learning tasks, such as the task of learning to play Atari using only pixels and the score [36]. For example, the pressure to “go somewhere new” in the pixel space of an Atari game might lead to jumping over obstacles, opening doors, picking up keys, etc.

Another interesting research question is whether agents that are rewarded for doing many things during their lifetime are more likely to discover any particular skills of interest. For example, Novelty Search does not explicitly reward agents for finding the end goal of a maze, but in fact produces agents that reach that goal far more often than traditional EAs that directly reward for getting closer to the goal [19]. Similarly, Innovation Engines attempt to maximize performance on a wide range of objectives; however, it was found that rewarding *all* of these stepping stones led to higher performance for any specific objective than could be obtained by an EA rewarding *only* that objective [27]. It could be the case that Curiosity Search might similarly enable agents to learn complex skills that would not otherwise be learned by traditional goal-oriented EAs or Novelty Search. For example, rewarding a child solely for their ability to do cartwheels may never result in that behavior being learned. However, encouraging a child to play and do new things—such as walking, running, doing handstands, and tumbling—may ultimately enable that child to discover and master cartwheels. It has been shown that for animals such as hyenas and birds, the expression of many diverse behaviors tends to improve performance on complex problem-solving tasks [10, 37, 38]. Curiosity Search explicitly encourages the performance of many different behaviors and may thus provide similar benefits.

Along a similar vein, many real-world skills are hierarchical in nature; for example, most organisms must learn to stand before they can walk, learn to walk before they can run, etc. The experiments in this paper did not examine a problem domain with hierarchical skills. For example, agents do not need to learn how to open a yellow door as a *prerequisite* to learning how to open a red door. It would therefore be interesting to see whether Curiosity Search might also aid the accumulation of hierarchical skills in the individuals it produces, such that agents build upon their existing skill sets to produce more complex skills over evolutionary time.

We have shown that Curiosity Search in combination with Novelty Search produces better generalists than either algorithm alone. It would be interesting to further investigate this phenomenon to understand the nature of this synergy and to ascertain whether Curiosity Search is subject to fitness deception, as we expect. We would also like to investigate whether Curiosity Search performs just as well when combined with diversity-promoting objectives other than Novelty Search.

Finally, the edict “go somewhere new” is only a proxy for the larger aim of Curiosity Search, which is to “*do* something new.” In particular, we hope to investigate how Curiosity Search performs with a more sophisticated way of determining intra-life novelty (e.g. as with Innovation Engines [27], by using a deep neural network that can identify new, interesting things).

## Conclusions

In this paper, we described how EAs tend to produce *specialists*, each possessing only a small subset of skills. It would also be helpful for EAs to be able to produce *generalists*, or agents possessing a large and diverse skill set. At first glance, one approach to produce generalists might be Novelty Search, because it encourages agents to do different things. However, upon reflection, it is clear that encouraging population diversity is not sufficient to produce generalists—specialists inherently do “different things” from one another and are thus rewarded by the algorithm. On a multi-skill maze exploration task, we empirically confirmed that specialists are indeed produced by the most common form of Novelty Search (which rewards agents for ending up in different places). However, we also showed that a different implementation of Novelty Search, which considers *all* of the actions within an agent’s lifetime, performs significantly better at producing generalists.

Additionally, we introduced a new algorithm called Curiosity Search that explicitly rewards agents for performing as many behaviors as possible. Because the automatic identification of new and increasingly complex behaviors is not yet technically feasible, we tested a simpler version of Curiosity Search which instead rewards agents for going to many different places. On the same multi-skill maze exploration problem, we showed that Curiosity Search is able to produce individuals that explore significantly more than either Novelty Search or a baseline algorithm designed to produced specialists. We also showed that Curiosity Search acquires the skills necessary to explore the domain and tends to acquire significantly more of these skills than even a lifetime-aware version of Novelty Search. Our results thus provide empirical evidence that Curiosity Search can produce generalists, albeit on a simple domain designed to highlight that property. In future work, we plan to further develop Curiosity Search to produce generalists in more complex environments and on more difficult tasks.

In addition to its own merits, we showed that Curiosity Search can be a useful helper objective for encouraging the production of generalists when combined with other algorithms. Further, we provided evidence that *any* algorithm that rewards intra-life behavioral novelty (e.g. Novelty Search with a trajectory-based behavioral characterization) may facilitate the production of generalists. Consequently, any problem in which generalists are desired might benefit from the inclusion of Curiosity Search (or a similarly-inspired algorithm) as an additional search objective.

Overall, we have shown that the motivating principle behind Curiosity Search—encouraging agents to continuously “do something new”—may serve as a powerful tool for producing generalists. Although the implementation described in this paper is quite simple, we believe that with continued development, Curiosity Search has the potential to become an effective instrument for producing autonomous agents that continually explore their environment, acquire new skills over the course of their lifetime, and are more capable of adapting to new environments and challenges.

## Supporting Information

S1 Fig Typical trajectories for all mazes (except for the Cross Maze, shown in Fig 4). From left to right, each row shows fifty *NS*_*E*_ trajectories overlaid to show population-level coverage, a single typical *NS*_*E*_ trajectory, a typical *CS* trajectory, and a typical *NS*_*T*_ trajectory. Each row shows data from the same run. Although Novelty Search readily explores the entire maze (left column), a single individual from *NS*_*E*_ tends to specialize (middle-left column), exploring less than the individuals produced by Curiosity Search (middle-right column). When Novelty Search is given a lifetime-aware behavioral metric (right column), it tends to produce individuals more akin to those of Curiosity Search. On some mazes (e.g. the Hard Maze and S Maze, second and third rows), *NS* and *CS* appear to perform equally well. However, Curiosity Search outperforms Novelty Search in aggregate, as shown in Fig 5, and is never beaten by Novelty Search in producing generalists.
(EPS)

S2 Fig Performance over time on all mazes (except for the Cross Maze, shown in Fig 7). The *populations* of each treatment explore the entire maze, but Curiosity Search produces *individuals* that explore more than those of other treatments.
(EPS)

S3 Fig Curiosity Search agents acquire and remember more door-opening skills than Novelty Search agents. The best-exploring agents produced by evolution (Fig 5) are placed in the four mazes shown in **(a)**, each of which is designed to test a single door-opening skill (the different colors/striping patterns for each door correspond to those in Figs 2 & 3). No further evolution is performed. Agents are given 250 time steps in each maze and receive scores based on the number of mazes in which they successfully open the door (up to 4; values are normalized to fall within [0, 1]). Each maze is intentionally small such that agents are always within each door’s opening range (i.e. no navigation is required to open the door). **(b)** Although the median differences are small (an expected result given the small range of possible scores), agents evolved via Curiosity Search (*CS*) have a significantly higher interquartile range than those of Novelty Search (*NS*_*T*_, *p* < 0.05 on three out of five mazes). In aggregate, both *CS* and the multi-objective combination of Curiosity Search with Novelty Search (*CN*_*T*_) are able to open more doors than *NS*_*T*_ alone (*p* < 0.001). Agents evolved via Curiosity Search methods appear better able to learn and remember how to open doors than Novelty Search or Target Endpoints (*TE*), providing further evidence that the doors represent distinct, non-trivial skills that Curiosity Search is better able to acquire.
(EPS)

S4 Fig Curiosity Search agents are able to acquire door-opening skills without any explicit fitness pressure to do so. The exploration results for the default Curiosity Search fitness function in this paper, which includes both tiles and doors (*CS* and *CN*_*T*_, *f* = *t* + *d*), are shown here for comparison. When doors are removed from the fitness function, leaving only the number of tiles visited in the fitness function (*CS*_*O*_ and *CN*_*TO*_, *f* = *t*, where the ‘*O*’ subscript denotes tiles *‘only’*), Curiosity Search still acquires door-opening skills at the same rate (*p* ≥ 0.05 for every maze and in aggregate). Although doors are not included in the fitness function for *CS*_*O*_ or *CN*_*TO*_, agents are still measured in this plot by the number of tiles explored and doors opened (for easier comparison to regular Curiosity Search agents). These results indicate that Curiosity Search does not need to directly reward the skills an agent should develop; it is enough for the algorithm to reward exploration alone.
(EPS)
