# Correction: Curiosity Search: Producing Generalists by Encouraging Individuals to Continually Explore and Acquire Skills Throughout Their Lifetime

**DOI:** 10.1371/journal.pone.0169313

**Published:** 2016-12-22

**Authors:** Christopher Stanton, Jeff Clune

The following information is missing from the Funding section: This study was supported by a National Science Foundation CAREER award (grant 1453549) to JC. The funders had no role in study design, data collection and analysis, decision to publish, or preparation of the manuscript.
